# Morphological convergence and adaptation in cave and pelagic scale worms (Polynoidae, Annelida)

**DOI:** 10.1038/s41598-021-89459-y

**Published:** 2021-05-21

**Authors:** Brett C. Gonzalez, Alejandro Martínez, Katrine Worsaae, Karen J. Osborn

**Affiliations:** 1 Department of Invertebrate Zoology, Smithsonian Institution, National Museum of Natural History, P.O. Box 37012, Washington, DC USA; 2 Molecular Ecology Group (MEG), Water Research Institute (IRSA), National Research Council of Italy (CNR), Largo Tonolli, 50, Pallanza, Italy; 3 Marine Biological Section, Department of Biology, University of Copenhagen, Universitetsparken 4, Copenhagen Ø, Denmark; 4 Monterey Bay Aquarium Research Institute, 7700 Sandholdt Road, Moss Landing, CA USA

**Keywords:** Marine biology, Molecular evolution, Phylogenetics, Taxonomy

## Abstract

Across Annelida, accessing the water column drives morphological and lifestyle modifications—yet in the primarily “benthic” scale worms, the ecological significance of swimming has largely been ignored. We investigated genetic, morphological and behavioural adaptations associated with swimming across Polynoidae, using mitogenomics and comparative methods. Mitochondrial genomes from cave and pelagic polynoids were highly similar, with non-significant rearrangements only present in cave *Gesiella.* Gene orders of the new mitogenomes were highly similar to shallow water species, suggestive of an underlying polynoid ground pattern. Being the first phylogenetic analyses to include the holopelagic *Drieschia,* we recovered this species nested among shallow water terminals, suggesting a shallow water ancestry. Based on these results, our phylogenetic reconstructions showed that swimming evolved independently three times in Polynoidae, involving convergent adaptations in morphology and motility patterns across the deep sea (*Branchipolynoe*), midwater (*Drieschia*) and anchialine caves (*Pelagomacellicephala* and *Gesiella*). Phylogenetic generalized least-squares (PGLS) analyses showed that holopelagic and anchialine cave species exhibit hypertrophy of the dorsal cirri, yet, these morphological modifications are achieved along different evolutionary pathways, i.e., elongation of the cirrophore versus style. Together, these findings suggest that a water column lifestyle elicits similar morphological adaptations, favouring bodies designed for drifting and sensing.

## Introduction

Pelagic annelids are largely understudied, with poorly known origins, an unrecognized diversity and delicate bodies. Their mystery lies in their original discovery, which in most cases, dates back several centuries and are based on single observations of often incomplete or damaged specimens^1^. Yet, annelids are not uncommon throughout the oceanic water column, the midwater, with several groups containing only holopelagic species, including Lopadorrhynchidae, Iospilidae, Typhloscolecidae, Tomopteridae^2^, Alciopini, and *Ctenophoricola*^3^. Among the obligate holopelagic groups, evolution has driven numerous morphological specializations, including well-developed eyes (Alciopini), transparent bodies (Tomopteridae), specialized feeding and reproductive modes (Lopadorrhynchidae, *Ctenophoricola*) and creative defensive strategies (Alciopini^4,5^; *Swima*^6^). Holopelagic species are found within several other annelid families too, and are assumed to have evolved by opportunistic colonization events within otherwise benthic lineages. Such examples include *Poeobius* and *Flota*, two unrelated holopelagic genera within the otherwise benthic Flabelligeridae^7^, *Swima* and *Teuthidodrilus* within Acrocirridae^8^, Alciopini and *Ctenophoricola* within Phyllodocidae^3^ and C*haetopterus pugaporcinus* Osborn, Rouse, Goffredi & Robison, 2007 within the tube dwelling Chaetopteridae^9^. Several other annelid representatives can be found in the water column, but distinguishing truly pelagic forms from late stage larvae, or species with periodic swimming has proven challenging given the limited amount of direct observations available. Countless records of “benthic” annelids being collected from the water column are known^2^, but much work remains before we understand the true significance of benthic–pelagic interactions in annelids. Holopelagic species described from otherwise benthic groups often exhibit morphological adaptations that are convergent with those seen in exclusively holopelagic clades^2^. Regardless of their origin, annelids using the water column as habitat have undergone shifts in their behaviour and posture, specifically with regards to motility and lifestyle^10^. Some holopelagic species appear to swim continuously (e.g., Alciopini), many alternating between active swimming and drifting (e.g., Tomopteridae, *Swima, Poeobius*, and *Flota*) and others only drifting as in C*haetopterus pugaporcinus*.

Whether exclusive to, or only periodic forays, annelids tend to have a particular set of morphological characteristics enabling them to survive in the water column habitat. Characteristics typically associated with water column use include elongate parapodia; combination of flattened, longer or more numerous chaetae (e.g.,* Lopadorrhynchus*); reduced musculature (e.g., Tomopteridae, Typhloscolidae, *Poeobius*); reduced (e.g., Lopadorrhynchidae, *Poeobius, Flota, C. pugaporcinus*) or greatly expanded number of segments (e.g., some Alciopini); augmented sensory appendages, including eyes (i.e., Alciopini or *Teuthidodrilus*), branchiae*,* cirri (e.g., acicular cirri in Tomopteridae, or dorsal cirri in *Gesiella* and *Pelagomacellicephala*); and transparent bodies or body parts (e.g., Tomopteridae, some Alciopini, *Poeobius, Flota*).

Scale worms (Aphroditiformia) are benthic predators employing either active or sit-and-wait foraging strategies^11^, yet, few behavioural studies have been conducted. Prior to ROV exploration, swimming scale worms were rarely documented, and while early midwater exploration recovered numerous representatives, the ecological significance of such findings were largely ignored^1,12^. Scale worms have been reported from the midwater since at least 1892^13^, however, it was not until the “Michael Sars” cruise of 1910 that polynoids were truly recognized for their water column forays^1^. With continued exploration, species previously thought to be exclusively benthic have been regularly caught above the bottom and even high in the midwater, often representing a large portion of the collected material from towed nets^1,2^.

Only three polynoid genera are described as being holopelagic, *Drieschia*^13^, *Podarmus*^14^ and *Drieschiopsis*^15^, but benthopelagicism is noted in several polynoid groups, including, but not limited to, Macellicephalinae and Admetellinae^12,16^*.* Little is known about Admetellinae, but Macellicephalinae are a prolific, obligate deep sea group, characterized by delicate elytra, long chaetae and elongated parapodia and dorsal cirri. These characters have been proposed to facilitate life in the water column^12^. None of the Macellicephalinae are described as being pelagic, however, two genera are known to exclusively inhabit water columns of inland anchialine caves, *Gesiella* and *Pelagomacellicephala*^17–22^.

Aphroditiformia is one of the most diverse groups within Annelida, yet remains represented by only a few exemplars in phylogenetic studies, especially for taxa occurring exclusively in difficult to access habitats such as the midwater and anchialine caves. While group specific analyses continue to provide valuable sequence and biogeographical information across understudied lineages (see Lindgren et al.^23^; Hatch et al.^24^), broad scope analyses remain limited, hindering understanding of scale worm evolution. In an attempt to rectify this, we investigated mitogenomics in members of Polynoidae across different environments, specifically midwater (i.e., *Drieschia*) and anchialine caves (i.e., *Gesiella* and *Pelagomacellicephala*). Our analyses included polynoid representatives from shallow water, midwater, deep sea vents, commensal/parasitic specialists and from anchialine caves. This is the first phylogenetic analyses to include the genus *Drieschia*, an elusive holopelagic scale worm genus with a previously unknown phylogenetic affinity. By including multiple genetically distant representatives that access the water column (i.e., *Branchipolynoe, Gesiella, Pelagomacellicephala, Drieschia*), we can now identify if swimming is a result of a single colonization event, or, if swimming and its associated behavioural and morphological adaptations occurred multiple times independently within the Polynoidae.

Herein, we use an integrative approach to characterize the genetics and the morphological characteristics of Polynoidae that live in the water column, incorporating mitochondrial genome analyses and phylogenetic comparative methods to describe the ecological drivers and morphological changes related to colonization of the water column.

## Results

### Mitochondrial genome characterization

**Figure 1:**
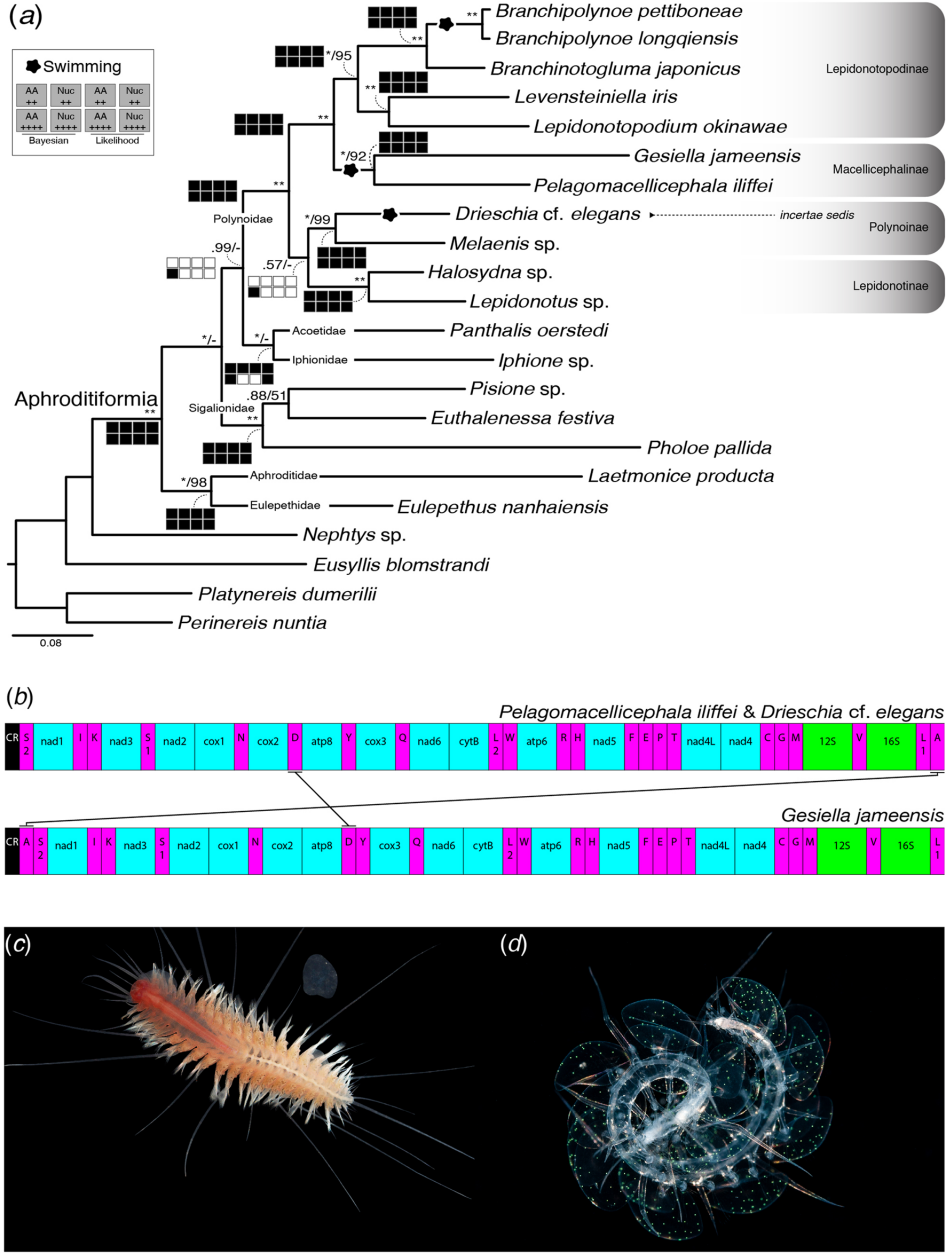
Mitogenomic analyses of Aphroditiformia. (**a**) Mitogenome relationships of Aphroditiformia based on the concatenated dataset of 13 protein coding genes translated into AA + 12S + 16S + 18S + 28S. Tree topology based on the Bayesian analysis (BA) of the dataset. Only nodal support above BPP = 0.5 and MLB = 50 are displayed. Nodes not recovered across analyses or with support lower than BPP = 0.5 and MLB = 50 are represented by a dash (–). Asterisks (*) denote BPP = 1.0 or MLB = 100. “Navajo rugs” indicate recovered (black squares) and unrecovered (white squares) nodes for each of the datasets across both BA and ML. Abbreviations: AA++, 13 PCGs translated into AA + 12S + 16S; AA++++, 13 PCGs translated into AA + 12S + 16S + 18S + 28S; Nuc++, 13 PCG (nucleotides) + 12S + 16S; Nuc++++, 13 PCG (nucleotides) + 12S + 16S + 18S + 28S. (**b**) Mitochondrial gene orders across the three newly generated mitogenomes. Gene order of *Pelagomacellicephala iliffei* and *Drieschia* cf. *elegans* follow that of the putative Polynoidae ground pattern. Black lines indicate the location and direction of gene rearrangements as indicated by the software CRExy^63^. Putative location of the control region (CR) is indicated by black filled rectangles, while fuchsia rectangles indicate tRNA’s, turquoise for PCGs and green for mt rRNAs. (**c**) Cave *Pelagomacellicephala iliffei.* Photo taken by Jørgen Olesen (**d**) Holopelagic cf. *Drieschia.* Photo courtesy of Linda Ianniello.

Nearly complete mitogenomes of swimming cave scale worms, *Pelagomacellicephala iliffei* Pettibone, 1985 and *Gesiella jameensis* (Hartman-Schröder, 1974), and holopelagic *Drieschia* cf. *elegans,* had lengths of 14,605 bp, 14,727 bp and 14,730 bp, respectively, including intergenic nucleotides (Supplementary Dataset 1). The control region was missing across each mitogenome, which we were unable to sequence within our resources. Each mitogenome contained 37 genes, including 13 PCGs, 2 mt rRNA- and 22 tRNA genes (Fig. 1b). All genes from the three mitogenomes are transcribed and encoded on the plus strand. Genes *nad4L* and *nad4* overlap by 4 bp in each. Generally, PCGs, tRNA’s and mt rRNA’s are all biased towards A and T, with negative AT- and GC-skews (Table 1). Supplementary Dataset 1 for details and exceptions.

**Table 1:** Genomic features for each of the three newly constructed mitogenomes.

	*Pelagomacellicephala iliffei*	*Gesiella jameensis*	*Drieschia* cf. *elegans*
**Mitogenome**			
Length (bp)	14,605	14,727	14,730
AT %	63.85	77.84	61.77
GC %	35.96	21.25	38.04
AT-skew	− 0.1512	− 0.0605	− 0.1541
GC-skew	− 0.3736	− 0.1572	− 0.2623
**Protein coding genes**			
Length (bp)	11,061	11,125	11,135
AT %	63.06	78.08	61.11
GC %	36.94	21.92	38.89
AT-skew	− 0.1954	− 0.1066	− 0.1968
GC-skew	− 0.4288	− 0.1849	− 0.3085
**tRNA**			
Length (bp)	1,430	1,407	1,451
AT %	69.37	81.24	64.44
GC %	30.63	18.76	35.56
AT-skew	0.0242	0.0481	− 0.0032
GC-skew	− 0.0776	− 0.0076	0.0039
**mt rRNA**			
Length (bp)	2.087	2.062	2.116
AT %	65.12	79.29	64.18
GC %	34.88	20.71	35.82
AT-skew	− 0.0522	0.1083	− 0.0442
GC-skew	− 0.2418	− 0.0913	− 0.1794

Additional information for individual genes can be found in Supplementary Dataset 1.

Every PCG in all three mitogenomes start with ATG start codon. Exceptions to this were found in *G. jameensis,* where genes *nad1*, *nad2*, *nad3*, *nad5* and *nad6* used TTG start codon. Stop codons were variable, either as TAA or the incomplete codon represented by a single T (Supplementary Dataset 1). Each mitogenome contained the typical metazoan set of 22 tRNA genes, including two tRNA serines and two tRNA leucines.

Gene order arrangement of the mitogenomes is illustrated in Fig. 1b, with colours indicating PCGs, mt rRNAs and tRNAs. Mitogenomes of *P. iliffei* and *D.* cf. *elegans* have identical gene orders, whereas *G. jameensis* has two rearrangements and one transposition. The nonsignificant rearrangement in *G. jameensis* involved repositioning *trnA* before *trnS2* and the transposition involved genes *trnD* and *atp8*, where *trnD* is no longer between *cox2* and *atp8* (Fig. 1b). Supplementary Figure S1 for gene order comparisons among all mitogenomes.

### Phylogenetic analyses

Our phylogenetic reconstructions were highly congruent between methods and datasets. All analyses found Aphroditiformia monophyletic (BPP = 1.0; MLB = 100). Navajo rug plots show the recovery (or lack) of select nodes between each dataset as compared to Fig. 1a (concatenated dataset of 13 PCGs translated into AA + 12S + 16S + 18S + 28S). See supplementary Figure S2 for tree topologies from the other dataset analyses.

Terminals corresponding to the aphroditiform families formed highly supported clades, yet many of them with low representation (Fig. 1a). However, relationships amongst the clades for each family varied by analyses (see Navajo rugs in Fig. 1a*;* Supplementary Figure S2). These variations included the position of Acoetidae and Iphionidae, forming the clade Acoetidae–Iphionidae in all but two analyses, and the position of Sigalionidae, forming the clade Sigalionidae–Polynoidae (BPP  ≥ 0.90; MLB = 56–75) in several analyses (Supplementary Figure S2). Aphroditidae and Eulepethidae always formed a well-supported clade sister to the remaining scale worms.

Terminals corresponding to the family Polynoidae were inferred as monophyletic in all analyses and representatives of the subfamilies Lepidonotopodinae, Macellicephalinae, Lepidonotinae and Polynoinae–*incertae sedis* being recovered with highly supported clades.

Anchialine cave species *P. iliffei* and *G. jameensis* formed a well-supported clade (BPP = 1.0; MLB = 92) in all analyses (Fig. 1a), sister to *Lepidonotopodium okinawae–B. pettiboneae* (BPP = 1.0; MLB = 95)*.* Together, these two clades formed the fully supported ‘deep sea clade’ (BPP = 1.0; MLB = 100) as in previous analyses.

The holopelagic *Drieschia* cf. *elegans* was recovered independent from all deep-sea lineages, forming a clade with the shallow water *Melaenis* sp. (BPP = 1.0; MLB = 99) in all analyses. This clade was recovered sister to *Lepidonotus* sp.–*Halosydna* sp. in the analyses using the 13 PCGs translated into AA + 12S rRNA + 16S rRNA + 18S rRNA + 28S rRNA, but with low support (BPP = 0.57). In all other analyses, the clade *Lepidonotus* sp.–*Halosydna* sp. was fully recovered, but sister to the clade containing subclades *Melaenis* sp.–*D.* cf. *elegans* and *P. iliffei*–*B. pettiboneae.*

Given the recovered relationships (Fig. 1a), three independent shifts into a swimming lifestyle were identified; the holopelagic *D.* cf. *elegans*; cave species *P. iliffei–G. jameensis*; and deep-sea species *Branchipolynoe longquiensis–Branchipolynoe pettiboneae.*

### Hypothesis testing

#### Substitution rates on mitochondrial protein coding genes

**Figure 2:**
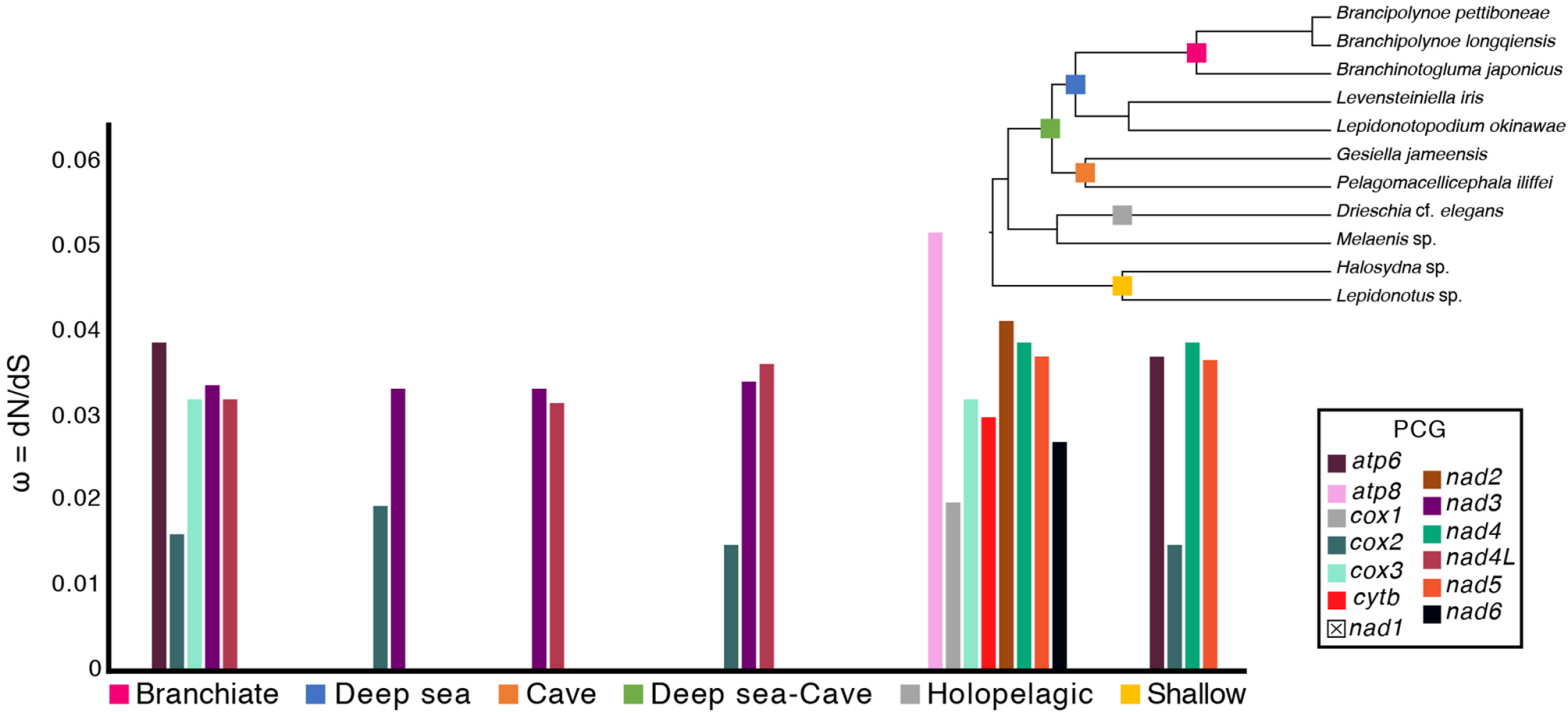
Estimated ratios (ω) of non-synonymous (d_N_) to synonymous (d_S_) substitution rates in Polynoidae for each of the individual 13 protein coding genes (PCGs) across selected nodes of the phylogeny in order to compare differences among habitats and lifestyles within Polynoidae. Missing bar graphs indicate that the log-likelihood (lnL) of the two-ratio model failed when compared against the lnL of the basic or null model (M0).

The significant non-synonymous/synonymous substitution ratios (ω) within Polynoidae revealed signatures of purifying (negative) selection (ω < 1) across each of the selected branches (Fig. 2; tree insert). Evaluation of single PCGs showed selection is largely gene specific among branches, with nearly all PCGs showing purifying selection, although with a select few showing less constraint than others (e.g.,* atp8, nad2, nad4*). Of the investigated branches, selection was most prevalent in the holopelagic branch, with eight PCGs showing evidence of purifying selection, followed by the branchiate branch (*Branchinotogluma–Branchipolynoe*) with five (Fig. 2). Purifying selection across select PCGs was also recovered across the other tested branches, although far less prevalent.

Of the selected branches, relaxed purifying selection was most noticeable in *atp8* (ω = 0.05197) and *nad2* (ω = 0.04149) of the holopelagic branch. Larger ω ratios were also recovered in *nad4* in both the holopelagic and shallow water branches. No discernible patterns were present across habitats for the PCGs. Evidence of selection was not detected in *nad1.*

#### Phylogenetic signal, PGLS and ancestral character estimations

As expected, phylogenetic signal inferred using Pagel’s λ and Blomberg’s K indexes were high for nearly all continuous characters examined, independently of the analyses (Table 2). Low phylogenetic signal was inferred for the “cirrophore length”, “parapodial length” and “parapodial width”. Continuous character reconstructions indicated a high variability in these traits, inferring multiple changes in size throughout polynoid evolution.

**Table 2:** Phylogenetic signal and significance values of selected traits.

	Non-ultrametric tree		Ultrametric tree	
	Phylogenetic signal	Mitogenome	Phylogenetic signal	Mitogenome
**Body length**				
Pagel’s λ	0.999	0.133	1.059	0.003
Blomberg’s *K*	0.788	0.367	1.004	0.122
**Body width**				
Pagel’s λ	0.999	0.161	1.057	0.042
Blomberg’s *K*	0.855	0.297	0.969	0.129
**Style length**				
Pagel’s λ	0.944	0.142	0.957	0.132
Blomberg’s *K*	1.040	0.041	0.989	0.095
**Cirrophore length**				
Pagel’s λ	0.882	0.294	0.836	0.370
Blomberg’s *K*	0.617	0.469	0.782	0.317
**Cirri length**				
Pagel’s λ	0.988	0.078	0.994	0.081
Blomberg’s *K*	1.108	0.035	1.079	0.040
**Ratio of cirri length: body length**				
Pagel’s λ	0.999	0.164	1.053	0.056
Blomberg’s *K*	1.148	0.036	1.018	0.105
**Parapodial length**				
Pagel’s λ	0.344	0.676	0.272	0.757
Blomberg’s *K*	0.657	0.338	0.747	0.339
**Parapodial width**				
Pagel’s λ	0.061	0.972	0.000	1.000
Blomberg’s *K*	0.737	0.303	0.893	0.226

Obtained from Pagel’s λ and Blomberg’s *K* statistics on non-ultrametric tree generated in RAxML using the concatenated dataset of 13 PCG (nucleotides) plus 12S rRNA, 16S rRNA, 18S rRNA and 28S rRNA.

Values close to zero for both λ and *K* statistics indicate low phylogenetic signal, therefore trait independence, while values close to one mean that traits are distributed as expected under a Brownian motion of trait evolution. Values greater than one indicate high phylogenetic signal (high trait similarity between related species).

**Figure 3:**
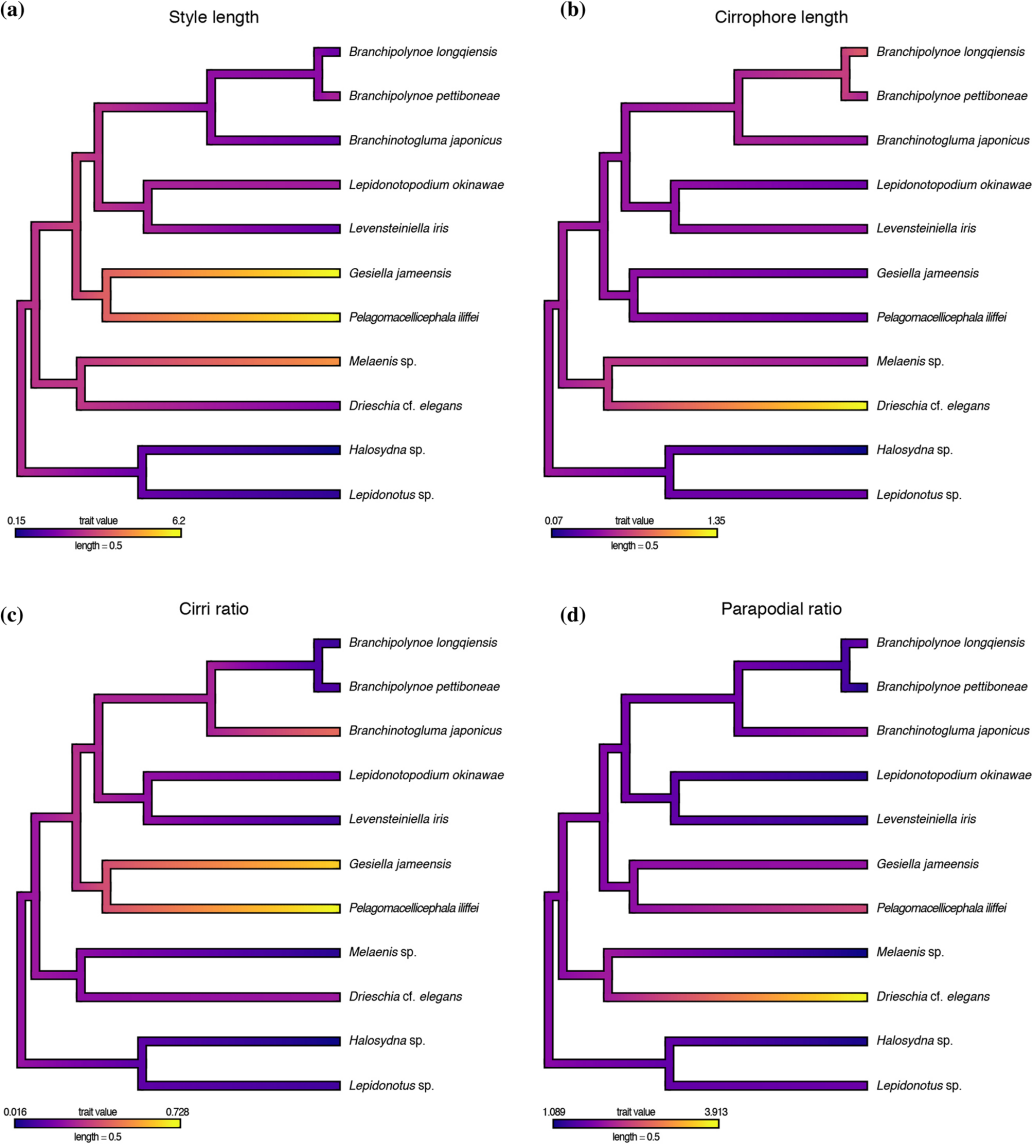
Phylogenetic Generalized Least Square (PGLS) analyses of measured parapodial traits in Polynoidae. (**a**) Continuous character “style length” mapped. (**b**) Continuous character “cirrophore length” mapped. (**c**) Mapped ratio of the “cirri length” to “body length”. (**d**) Mapped ratio of the “parapodial width” to “parapodial length”. Branch colours correlate to measured lengths of selected traits, bright colours (i.e., yellow) indicate large measured values while dark colours (i.e., indigo) represent short measured values.

Our phylogenetic generalized least-squares (PGLS) analyses showed a significant relationship between behavioural and morphological variables (Table 3). Specifically, “cirri length” (cirrophore + style) and ratio of “cirri length: body length” were significantly explained by “swimming”, whereas “cirri length”, “style length” and ratios of “parapodial length: parapodial width” and “style length: body length” were significantly explained by “drifting” behaviours. Continuous character tracing showed different morphological strategies for elongation of the dorsal cirri; elongating the style in *Pelagomacellicephala* and *Gesiella* (Fig. 3a), or the cirrophore in *Drieschia* (Fig. 3b). The effect of “body length” was expected; larger animals had larger cirri and parapodia.

**Table 3:** Results from the phylogenetic generalized least squares (PGLS) analyses of morphology correlated to the behaviours of “swimming” and “drifting”.

	PGLS	
	Variable	*p*MCMC
**Swimming**		
Cirri length	“body length”	0.0088**
	“swimming”	0.0146*
Cirri length: body length	“swimming”	0.0269*
Parapodial length	“body length”	0.0027**
	“swimming”	0.1819
Parapodial length: parapodial width	“body length”	0.1169
	“swimming”	0.6509
Style length	“body length”	0.9871
	“swimming”	0.4579
Style length: body length	“swimming”	0.0558
Cirrophore length	“body length”	0.7778
	“swimming”	0.0790
Cirrophore length: body length	“swimming”	0.9919
**Drifting**		
Cirri length	“drifting”	0.0031**
	“body length”	0.0504
Cirri length: body length	“drifting”	0.0072**
Parapodial length	“body length”	0.0057**
	“drifting”	0.5562
Parapodial length: parapodial width	“body length”	0.2389
	“drifting”	0.0488*
Style length	“body length”	0.1204
	“drifting”	0.0059**
Style length: body length	“drifting”	0.0082**
Cirrophore length	“body length”	0.2970
	“drifting”	0.8525
Cirrophore length: body length	“drifting”	0.4350

Based on our ancestral estimations of discrete characters, Polynoidae were non-swimming, non-drifting and possessed eyes. Our analyses estimated many transformations for the character swimming, while the capability of drifting evolved only twice, in the clades *Pelagomacellicephala–Gesiella* and *Drieschia–Melaenis* (though *Melaenis* is a non-drifting species). Eye loss occurred once in the clade *Pelagomacellicephala–Branchipolynoe.*

## Discussion

### Mitogenomic relationships in Aphroditiformia

Mitogenomes were recently explored for the first time in Aphroditiformia to test next generation sequencing techniques^25^, spanning the six recognized families and recovered findings consistent with those using fewer genetic markers (i.e., Norlinder et al.^26^; Gonzalez et al.^27^). Our results are largely congruent with previous studies, recovering a monophyletic Aphroditiformia consisting of six well supported family level clades (Fig. 1a). Our analyses however went beyond Zhang et al.^25^, adding additional mitogenomes, examining all available data in four different ways/datasets (Fig. 1a) and is the first study to phylogenetically place the holopelagic *Drieschia.*

In our analyses, the deeply nested clade Aphroditidae–Eulepethidae was always recovered sister to all remaining families. Sigalionidae was also recovered deeply positioned and included representative members of Pholoinae and Pisioninae (Fig. 1a). Between the four datasets, family level relationships varied in some analyses when compared to previous findings. Interestingly, our Polynoidae–Sigalionidae relationship was also recovered by Struck et al.^28^ and Zrzavý et al.^29^, but was attributed to lack of representation across aphroditiform families. As genes and taxa have increased, analyses have more often recovered Acoetidae and Iphionidae sister to Polynoidae, independent of Sigalionidae^25–27^. Variability in the position of Acoetidae and Iphionidae is not surprising, as this too was found in Gonzalez et al.^27^ between datasets. Our observed Polynoidae–Sigalionidae relationship could be an artefact of low taxonomic representation among the other families, or, it may be due to increased genetic similarity between taxa as datasets become progressively complex. Further analyses using more comprehensive datasets across each family are needed to unravel these conflicting hypotheses, nevertheless, both families represent the largest clades within Aphroditiformia, sharing many putatively homologous features, having widespread distributions and exhibiting multiple colonization events across marine habitats.

Mitochondrial gene order is still considered conserved in many groups, possibly because mitogenome data remains sparse. In Annelida, this speculation has led to the suggestion of a common ground pattern across Errantia and Sedentaria^30–32^. However, recent increases in mitogenome sampling across phyllodocid lineages (i.e., Syllidae, Aphroditiformia) showed that mitochondrial gene orders are more variable than previously anticipated^25,30,33,34^. In Aphroditiformia, Zhang et al.^25^ recovered six different mitochondrial gene orders from their sampling of 16 scale worms, four being from within Polynoidae. While no underlying ground pattern was identified, based on our new mitogenomes, a putative ground pattern appears to exist for Polynoidae, Iphionidae and Acoetidae, differing only slightly from other scale worm families. Both *P. iliffei* and *D.* cf. *elegans* conform to this putative Polynoidae ground pattern (Fig. 1b), and to a large extent, so does *G. jameensis,* differing only by the position of two tRNAs, which are generally more labile^34^. Similarly, this Polynoidae ground pattern was also found in the polynoid *Polyeunoa laevis* McIntosh, 1885, adding validity to the idea of a conserved gene order ground pattern in Polynoidae^35^. Truly labile mitogenomic architecture was only present in the branchiate commensal deep sea *Branchipolynoe* and *Branchinotoguluma* and the hydrothermal vent inhabiting *Lepidonotopodium* and *Levensteiniella*^25^*.* Aguado et al.^30^ proposed that genetic drift sustained from bottleneck events were responsible for similar highly divergent gene orders in Syllidae, especially in the gemmiparous *Ramisyllis* and *Trypanobia*. It is equally likely that adaptive processes resulting from environmental pressures are also responsible for variable gene orders seen in branchiate polynoids.

Deep sea polynoids in Zhang et al.^25^, together with *Pelagomacellicephala* and *Gesiella* (this study) made up a ‘deep sea clade’ consisting of Macellicephalinae and the recently erected Lepidonotopodinae taxa (previously all Macellicephalinae; see Hatch et al.^24^). Within the ‘deep sea clade’ there are three different gene orders, showing that closely related taxa are capable of highly variable gene arrangements. While bottlenecks and habitat differences may be responsible for mitochondrial gene orders in some annelid groups, our findings suggest that habitat shifts are not equally driving mitochondrial gene rearrangements. We find conserved gene orders in scale worms from both anchialine caves and the midwater despite both habitats subjecting inhabitants to strong environmentally driven selective pressure, as shown by their behavioural shifts and morphological transformations (Fig. 3a–d)^36–39^. Zhang et al.^25^ found significant differences between shallow- and deep-living polynoids, indicating purifying selection. While purifying selection was most prevalent in select PCGs of the holopelagic clade, we found no significant differences, or indication of strong purifying selection in one habitat over another, likely due to our increased taxon sampling. This lack of significant difference in selection suggests that nonsynonymous changes in polynoid mitogenomes are relatively conserved across habitats and species (Fig. 2). However, given the diversity and marine ubiquity of polynoids, it is likely that stronger purifying selection will be found in select groups, as well as additional gene rearrangements to the putative polynoid ground pattern. Evidence to this already exists in Sigalionidae, given the different gene orders between the three taxa sampled^25^, further suggesting that all Aphroditiformia, and Annelida, will exhibit more mitochondrial gene variability than previously expected.

### Behavioural adaptations

Behavioural adaptations associated with colonization of the water column remain uncertain in annelids because of the challenges associated with accessing the habitat and specimen rarity. We maintain the definition of holopelagic animals as those that live their entire lives within the oceanic water column, being distinct from those that permanently inhabit the water column in enclosed environments such as caves. However, based on behavioural observations, we employ the term ‘swimming’ to define scale worms that sink quickly upon cessation of active swimming, and ‘drifting’ for those that, when they stop actively swimming, remain suspended in the water column without sinking appreciably. *Drieschia, Gesiella* and *Pelagomacellicephala* all represent drifting taxa, while *Branchipolynoe* represents a swimming taxon. The remaining taxa are presumed to swim only for brief periods of time when disturbed. Our PGLS analyses (Table 3) found both “cirri length” and “cirri length: body length” were significantly correlated to “swimming”, and thus can be linked to polynoids accessing the water column from the benthos. In polynoids designated here as “drifting”, PGLS analyses showed “style length” and “cirri length” were significantly correlated to this behaviour. Interestingly, the ratio of “parapodial length: parapodial width” was also correlated to drifting, suggesting further specializations have occurred during the transition from the benthos to permanent use of the water column. Using continuous character tracing, we determined that based on behaviour, “drifting” equally favours different ways to achieve elongation of the dorsal cirri—either by elongation of the style or elongation of the cirriphore (Fig. 3a–c).

Swimming without drifting is seen in the commensal *Branchipolynoe*, which accesses the water column to travel between deep sea mussel beds within which they feed and take shelter^40^. *Branchipolynoe* have broad, relatively short parapodia and dorsal cirri given their body size (Fig. 3d). This parapodial morphology is likely not ideal for prolonged periods in the water column, as shorter parapodia are less effective paddles^41^ and shorter sensory structures would limit their three-dimensional sensory capabilities, yet aid in their mobility within the confines of their host mussels. Episodic swimming events also occur in the non-commensal shallow water species *Bylgides sarsi* (Kinberg in Malmgren, 1866) and *Harmothoe imbricata* (Linnaeus, 1767) when forced^42–44^, however, previous analyses^39^ showed no morphological adaptations to a water column lifestyle. While it is unknown to what extent the other tested branchiate (i.e., *Branchinotogluma*) or deep-sea groups (i.e., *Levensteiniella* and *Lepidonotopodium*) swim, their parapodial morphology suggests it would be highly similar to that of *Branchipolynoe.*

Prior analyses focusing on anchialine cave genera showed an elongation of the dorsal cirri, specifically the dorsal style (referred herein as “style length”), was correlated to swimming and inhabiting caves. It is likely that an elongated style provides a better means of spatial acuity and tactile detection. Having a dataset that now includes holopelagic and additional swimming species (Fig. 1a), we confirm that “cirri length” is correlated with “swimming”, but now additionally show that longer cirri are also correlated with “drifting” (Table 3). The specifics of morphological elongation vary based on genera and habitat (Fig. 3a,b). Both *Pelagomacellicephala* (Fig. 1c) and *Gesiella* are cave genera living in total darkness and exhibit elongation of the sensory portion of the cirri, the dorsal styles (“style length”), but show little (if any) modification to the cirrophore (muscular base of the cirrus). By only elongating the style, cave genera add length without bulky muscular components. When drifting in the water column, their cirri are spread out around the body, presumably to create the broadest possible 3D sensory field. In contrast, the holopelagic *Drieschia* (Fig. 1d) shows pronounced elongation in the muscular cirrophore. This likely enables the animals to orient their sensory appendages in the water column, a habitat with greater water movement and larger periodic disturbances than in cave habitats (Fig. 3b).

In polynoids, a holopelagic lifestyle has evolved in at least three genera, *Drieschia, Podarmus* and *Drieschiopsis* (latter two not available for this study). *Pelagomacellicephala* and *Gesiella* are not considered pelagic because caves lack a pelagic zone, yet these genera continuously inhabit the water column. *Drieschia’*s transparency (Fig. 1d) differs from the cave species (Fig. 1d) and is likely an adaptation to avoid visual predators in a habitat with nowhere to hide. Common midwater camouflage adaptations include transparency, mirrors, pigmentation, antireflective films and counterillumination^45,46^. The optically featureless midwater necessitates morphological and behavioural adaptations to avoid detection by predators^47^. Behavioural mimicry has received little attention in the midwater because of the limited behavioural observations available, but is likely to be used often to avoid predators and is seen in both vertebrates and invertebrates^10^. For example, animals with elongate body forms, such as fish and squid, have been observed to shapeshift when disturbed or threatened, becoming rounded or coiled, possibly to resemble unpalatable, stinging medusae^10^. The extent of such behaviours are poorly documented, but are known to also occur in a variety of invertebrates, like appendicularians, chaetognaths and annelids^4^. Tomopterids (Annelida), fast, agile swimmers capable of escape, displays a distinctive curling behaviour when agitated, interpreted as a form of Batesian mimicry given that their primary predator avoidance is their transparency and speed^10,48^. Similar to tomopterids*,* the holopelagic *Drieschia* (pers. comm. Linda Ianniello) also exhibits curling behaviours, positioning their outstretched cirrophores between inflated elytra, with styles projecting outwards (Fig. 1d). Blackwater divers regularly observe *Drieschia-*like scale worms drifting in the water column in this curled posture, uncurling when disturbed. This posture gives resting *Drieschia* an uncanny resemblance to medusae and may be a behavioural adaptation to midwater lifestyles. Curling behaviours are completely unknown in *Branchipolynoe* and cave genera, both living in habitats where visual predators are scarce and stinging medusae are not prevalent. Thus, we suggest that curling behaviours in holopelagic scale worms are a form of mimicry used in the midwater to visually trick potential predators.

Robison^10^ suggested that mesopelagic animals capable of behaviourally altering their appearance to predators often lacked bioluminescence as a way of camouflaging their silhouettes. However, *Drieschia* has strongly fluorescent cells in their elytra, which typically indicates bioluminescent capabilities, similar to other polynoids with bioluminescent elytra^49^. Additionally, all members of Tomopteridae emit bioluminescent light^48,49^ and have also been observed “curling”. It should be noted though that tomopterids eject bioluminescent mucus from the tips of their parapodia, which is unlikely to be used as a form of camouflage, but instead act as a “burglar alarm”. Consequently, in the case of epi-, meso- and holopelagic polychaetes, multiple strategies to avoid visual predators are employed.

As raptorial feeders, nearly all scale worms are visually oriented benthic predators relying on sensory and visual cues, as well as sighting distance to get within striking range without being detected^11,40,47^. For cave *Pelagomacellicephala* and *Gesiella,* the scarcity of both visual predators and light frees them from constraints on behaviour and morphology, permitting cruising predation without the need of camouflage or mimicry^40,50^. However, in the pelagic realm, where visual predators are abundant, active foraging is more complex. Unlike other polynoids, *Drieschia* has adapted its crypsis tactics to include visual mimicry (curling), transparency and possibly counterillumination. No other polynoid is known to utilize so many concurrent crypsis strategies.

### Morphological adaptations

The success of scale worms across marine environments can be attributed to their capacity to modify suites of morphological characters or their morphological plasticity. The ability to swim may have been a vital component of the flexibility of scale worm evolution. Our recovery of higher than expected probabilities for “swimming” in ancestral reconstructions provide evidence that all scale worms are capable of accessing the water column, a finding that is corroborated by early ecological records^1^. Based on our phylogenetic reconstructions for Polynoidae (Fig. 1a), swimming lifestyles have evolved at least three times, with the ability to drift arising independently twice (Fig. 1a). Evolution of drifting has each time selected for hypertrophy of the dorsal cirri. However, we interpret the elongation of the dorsal cirri in *Pelagomacellicephala–Gesiella* compared to *Drieschia,* as separate autapomorphies and not homoplasy due to the difference in how elongation is accomplished in each.

Cave inhabiting *Pelagomacellicephala* and *Gesiella* are related to strictly deep-sea species, meaning they share common ancestry and thus have inherited traits of anophthalmia and lack of pigmentation^27,40,51^. These traits together are referred to as darkness syndrome—the sharing of similar morphological and biological traits between obligate deep sea and subterranean fauna^52^. The connection between the deep sea and caves was proposed as early as 1894^52^, and has now been shown across several lineages, including decapods (e.g.,* Munidopsis*) and annelids^27,53–56^. In contrast, *Drieschia* lacks all traits associated with darkness syndrome (transparency is quite different than lack of pigmentation), and is nested within shallow benthic species. Based on its phylogenetic position and lack of troglomorphic traits, we see the elongation of the dorsal cirri as a form of convergent evolution.

*Drieschia* was consistently recovered sister to *Melaenis*, a shallow benthic, non-swimming clade possessing coloration and eyes. This suggests that *Drieschia* evolved from a shallow water ancestor, retaining only the presence of eyes. Unlike most, *Drieschia* appears perfectly adapted for the midwater (Fig. 1d), having a nearly transparent body and reduced musculature. The elytra are also transparent and with luminescent cells, being held well above the dorsal body surface, possibly aiding in buoyancy during drifting*.* While bioluminescence is a common defensive strategy among pelagic animals^57^, in *Drieschia,* it is likely an inherited trait from shallow water ancestors. Bioluminescence is known in at least nine, non-swimming benthic polynoid genera, and is used to confuse predators during escape^58^. Interestingly, luminescence is completely lacking in cave polynoids and unknown in most deep-sea groups.

**Figure 4:**
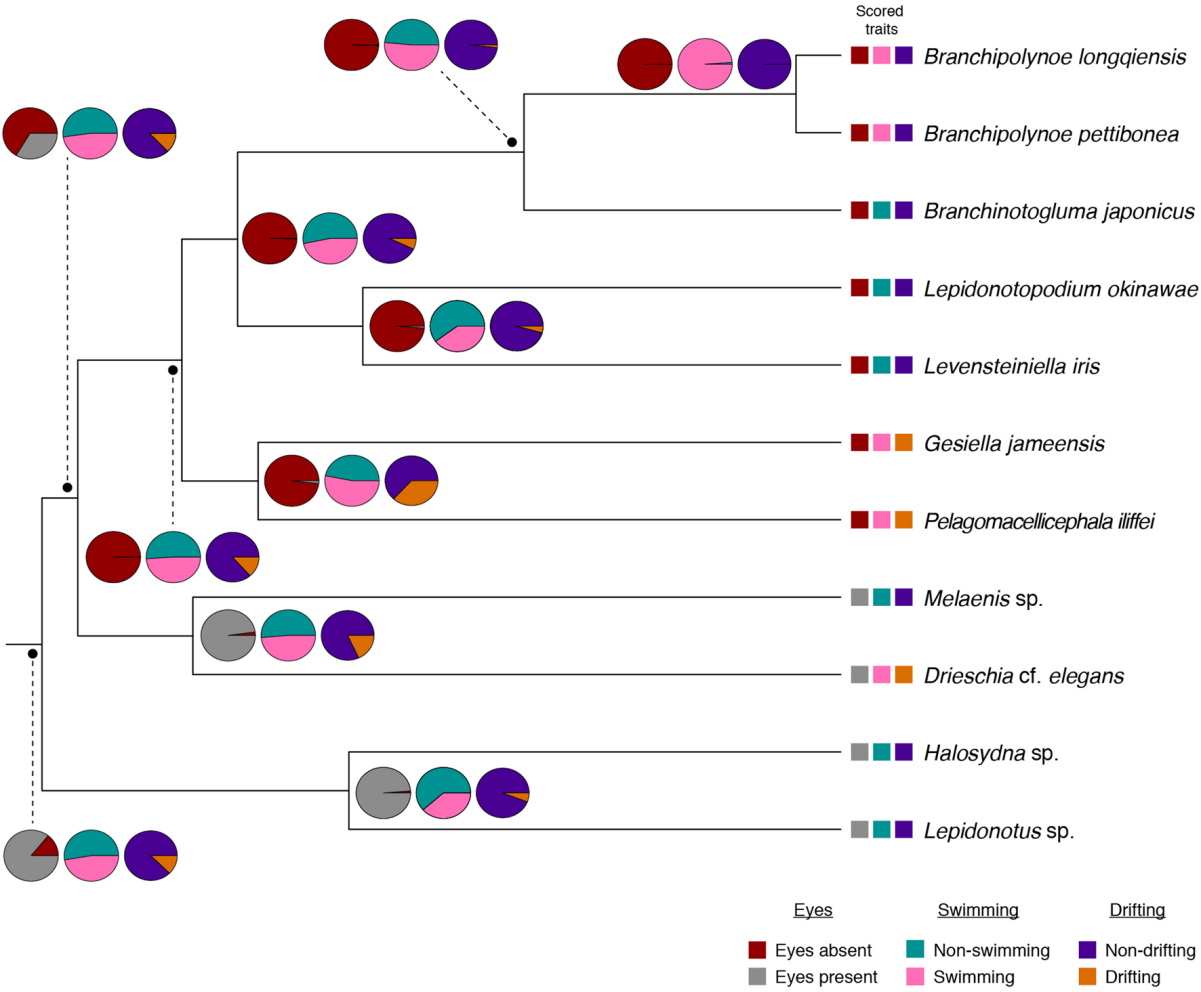
Reconstructed ancestral character estimations for Polynoidae. Coloured squares at branch tips correlate to scored traits for each taxon. Colour-coded pie charts reflect the overall probability of that character occurring in the ancestor of that particular node. Scored traits include “eyes” (absent/present), “swimming” (non-swimming/swimming) and “drifting” (non-drifting/drifting).

“Eyes” are highly variable in polynoids, with nearly all radiations into lightless environments showing reduction of eyes, consistent with darkness syndrome (Fig. 4). Cave genera, *Branchipolynoe* and all other included deep-sea taxa (i.e., *Branchinotogluma, Lepidonotopodium, Levensteiniella*) lack eyes. However, *Drieschia* (holopelagic) retains the characteristic polynoid eye morphology, likely necessitated by interactions occurring within the midwater. Eyes are also prevalent in other pelagic annelids. Most notably, Alciopidae, with their greatly enlarged, lensed eyes and thin, long, often transparent bodies^1^. Likely also evolved from a shallow water ancestor, alciopids are only known from the epi- and mesopelagic zones, retaining their eyes, as have their sister group, the newly described *Ctenophoricola*^3^. Similarly, lopadorrhynchids, tomopterids and iospilids have eyes, suggesting that eyes are an important feature for pelagic annelids.

## Concluding remarks

As with previous investigations, mitogenome analyses continue to recover evidence of cave and deep-sea relationships, recovering a fully supported ‘deep sea clade’. This is the only analyses to phylogenetically place the holopelagic *D.* cf. *elegans,* showing shallow water ancestry and thus a novel route to a holopelagic lifestyle. This is the first comprehensive dataset to investigate adaptations to life in the water column across habitats within Polynoidae using multidisciplinary approaches of mitogenomics and phylogenetic comparative methods. Our phylogenetic analyses show that swimming has evolved at least three times in Polynoidae, within the deep-sea *Branchipolynoe* and in both cave and holopelagic species. Based on PGLS results, swimming radiations displayed strong evidence of behavioural adaption in response to ecological constraints of their environment, with strong selection in drifters favouring hypertrophy of the dorsal cirri. Specifically, the holopelagic *Drieschia* has adapted to the water column by elongating their cirrophores to facilitate mimicry and increase manoeuvrability of their sensing cirri, while in cave polynoids, elongation is occurring in the styles. Together, these morphological adaptations reveal both similarities and differences in morphology and behaviour that allow scale worms to survive in the water column. These novel observations of the holopelagic *D.* cf. *elegans* drifting curled in the water column with dorsal cirri spread suggests they are employing a form of visual mimicry by resembling unpalatable animals. While lifestyle modifications are only known and tested in the four swimming groups that were available for this study, our ancestral character reconstructions suggest that swimming ability is likely common across Polynoidae, and more prevalent than previously recognized. Regardless of our distinction between ‘swimming’ and ‘drifting’, there is a wide range of unknown activities likely to impact the degree of specialization for life in the water column. In situ ocean exploration (see https://youtu.be/yrlSmxG5yZY) continue to show scale worms are more capable of swimming than currently recognized, emphasizing the fact that there remains much to learn about scale worms’ use of the water column.

## Methods

### Descriptive analyses of mitochondrial genomes

Individuals of *G. jameensis* and *P. iliffei* (Fig. 1c) were collected by scientific cave divers in October 2018 (Lanzarote, Canary Islands, Spain) and January 2019 (Turks and Caicos Islands), respectively (for collection site specifics, see Martínez et al. 2016; Gonzalez et al. 2020). *Drieschia* cf. *elegans* (Fig. 1d) was collected in February 2018 (off Cabo Verde Islands) from the *R/V Poseidon* using a multinet—max trawl with 4 mm mesh (15.308, − 25.056; 0–65 m depth; 2125 h; 25 Feb 2018—collector K.J. Osborn on GEOMAR expedition POS520)*.* Collection permits were obtained from each host country prior to carrying out any collection activities. Sampling of *Gesiella* was carried out under Permit 1298973 from Consejería de Medio Ambiente of the Government of Lanzarote (Canary Islands, Spain). From the Turks & Caicos Islands, *Pelagomacellicephala* was collected under Scientific Research Permit (SRP) No.: 18-12-01-23 issued through the Department of Environment and Coastal Resources (DECR) to Thomas M. Iliffe. Collections of *Drieschia* from Cabo Verde were granted under Autorização N°14/GP-CA.AMP/2017 issued through the Agência Maritima e Portuária and the Ministério da Agricultura e Ambiente to Henk Jan Hoving.

#### DNA extraction and Sanger sequencing

Genomic DNA (gDNA) was successfully obtained from tissue of a single dissected parapodia in *P. iliffei* and *D.* cf. *elegans,* stored in alpha numeric tubes preloaded with 150 μL of M2 buffer (AutoGen) at 4°C, and later extracted using the AutoGenprep 965 according to the manufacturer’s protocols. For *G. jameensis,* gDNA was obtained from tissue pieces of a single specimen using the Qiagen DNeasy Blood & Tissue Kit following the manufacturer’s protocol (Qiagen Inc., Valencia, CA, USA). Aliquots of gDNA were amplified using polymerase chain reactions (PCRs), targeting cytochrome oxidase I (COI) and 16S ribosomal RNA (16S rRNA) to be used as scaffolds for mitogenome assembly (see Harasewych et al.^59^ for detailed protocol). Remaining gDNA was used to construct gDNA libraries for Illumina sequencing. Methods and protocols for construction and annotation of mitogenomes are briefly summarized below; see Harasewych et al.^59^ for details.

#### Library construction and Illumina sequencing

Genomic DNA was visualized on a 1.5% agarose gel and quantified using a Qubit dsDNA HS Assay Kit (ThermoFisher, Pittsburgh, PA, USA). After quantification, gDNA was sonicated using the Covaris ME220 with microtube-50 AFA fibre screw-caps (Covaris, Woburn, MA, USA), targeting 350 base pairs (bp) fragments. Sonicated gDNA was cleaned using Kapa Pure Beads (KAPA Biosystems, Wilmington, MA, USA) at a ratio of 0.9× beads to sample, targeting fragments larger than 250 bp. Further validation of the gDNA size was performed using an Agilent 2200 TapeStation (Agilent, Santa Clara, CA, USA). Total gDNA libraries were prepared using the NEBNext Ultra II DNA Library Prep Kit for Illumina, in combination with the NEBNext Multiplex Oligos for Illumina (New England BioLabs, Ipswich, MA, USA). A 4 nM library concentration was denatured for clonal amplification and sequenced on an Illumina MiSeq (Illumina, San Diego, CA, USA) with MiSeq Reagent Kit v3. All sequencing was performed at the Laboratories of Analytical Biology, National Museum of Natural History, Smithsonian Institution, Washington, D.C., USA.

#### Genome assembly, annotation and composition

Mitogenomes were assembled using Trinity 2.6.6 with adaptor trimming by Trimmomatic. Assemblies was run on “Hydra”, the Smithsonian Institution High Performance Cluster (SI/HPC). Mitochondrial elements were annotated using MITOS^60^ and the ORF finder in Geneious 11.1.5.

The AT and GC skew patterns were calculated for all newly assembled mitogenomes according to the formulas AT-skew = (A − T)/(A + T) and GC-skew = (G − C)/(G + C)^61,62^. Skewness was independently calculated for each mitogenome, across each gene (Supplementary Dataset 1), as well as across concatenated gene blocks representing the 13 protein coding genes (PCGs) and the entire mitogenome (Table 1). Characterization of nucleotide and codon usage bias was calculated using Geneious 11.1.5.

#### Comparison of mitochondrial gene order

The software CREx^63^ was used to conduct pairwise comparisons of the mitogenomes of *P. iliffei, G. jameensis* and *D.* cf. *elegans.* CREx inferred the most plausible scenarios for gene rearrangements based on common intervals. The inferred gene order was compared to other species belonging to the family Polynoidae in the dataset.

#### Substitution rates on mitochondrial protein coding genes

Previous phylogenetic studies highlighted differential positive selection between deep sea and shallow water polynoids^25^. In order to compare whether the level of positive selection in cave and holopelagic polynoids resembles that of deep sea or shallow water species, we estimated the ratio (ω) of non-synonymous (d_N_) to synonymous (d_S_) substitution rates in Polynoidae across the 13 protein coding genes (PCG). To investigate if positive selection occurred on specific branches across the different PCG, branch models were used in order to compare differences among habitats and lifestyles across polynoids (Fig. 2). All analyses were implemented using the program EasyCodeML^64^, which is an interactive visual tool based on CODEML as part of the PAML 4.8.2 package^65,66^. Specifically, the one-ratio model (M0; all branches have the same ω values) was compared against the two-ratio model (one ω ratio for branches of interest ‘foreground’ and the other for the background). The log-likelihood (lnL) of the two-ratio model was compared against the lnL of M0, which is used to test if the selected branches evolve with different rates than background branches. Log-ratio tests (LRT) were calculated to compare the two models and to test for significance (Supplementary Dataset 1).

### Phylogenetic analyses

Three newly obtained mitogenomes were assembled and aligned against the 15 known scale worm mitogenomes (Table 4) (see Zhang et al.^25^). Alignments of PCGs were performed on the TranslatorX server^67^ using MAFFT. Default parameters were allowed for PGCs^68^, while a E-INS-I iterative refinement algorithm, which is optimized for sequences with multiple conserved domains and long gaps^69^, was selected for mitochondrial (12S rRNA and 16S rRNA) and nuclear (18S rRNA and 28S rRNA) ribosomal RNA genes^68^, together with the ‘nwildcard’ option, which does not designate missing data as gaps. All aligned sequences were post-treated using Gblocks v.0.91b^70^ (see Supplementary Dataset 1), removing ambiguously aligned positions using the options ‘allow smaller final blocks’, ‘allow gap positions within the final blocks’ and ‘allow less strict flanking positions’. Prior to phylogenetic analyses, each PGC alignment was visually checked for stop codons and reading frames using Mesquite v.3.51^71^.

**Table 4:** List of all taxa used for this study, including collection locality and GenBank accession numbers.

Family	Species	Collection locality	Mitogenome	18S-28S	Incomplete genes
Acoetidae	*Panthalis oerstedi*	Trondheimsfjord, Norway	KY753832	KY753846	
Aphroditidae	*Laetmonice producta*	Shag Rock, Antarctica	KY753833	KY753853	
Eulepethidae	*Eulepethus nanhaiensis*	Daya Bay, China	KY753834	KY753850	28S rRNA
Iphionidae	*Iphione* sp.	Pak Sha Wan, Hong Kong	KY753835	KY753852	
Polynoidae	*Branchinotogluma japonicus*	Sakai Vent Field, Okinawa Trough	KY753824	KY753841	28S rRNA/trnG
	*Branchipolynoe longquiensis*	Southwest Indian Ridge, Dragon Vent Field	KY753826	KY753847	28S rRNA
	*Branchipolynoe pettiboneae*	South China Sea	KY753825	KY753840	
	***Drieschia* cf.* elegans***	Cape Verde	**MW794259**	**MW794262**	
	***Gesiella jameensis***	Lanzarote, Canary Islands, Spain	**MW794260**	**MW794263**	
	*Halosydna* sp.	Yung Shue O, Hong Kong	KY753830	KY753845	
	*Lepidonotopodium okinawae*	Sakai Vent Field, Okinawa Trough	KY753828	KY753842	28S rRNA/trnL1/trnaA
	*Lepidonotus* sp.	Clear Water Bay, Hong Kong	KY753831	KY753851	
	*Levensteiniella iris*	East Scotia Ridge	KY753827	KY753848	
	*Melaenis* sp.	Tai Tam, Hong Kong	KY753829	KY753849	28S rRNA
	***Pelagomacellicephala iliffei***	Middle Caicos, Turks and Caicos Islands	**MW794261**	**MW794264**	
Sigalionidae	*Euthalenessa festiva*	Cook’s Bay, Mo’orea	KY753837	KY753839	28S rRNA
	*Pholoe pallida*	Kristineberg, Sweden	KY753838	KY753843	cox2/nad2/nad4/nad5
	*Pisione* sp.	Baja California, Mexico	KY753836	KY753844	28S rRNA
Nereididae	*Perinereis nuntia*	–	JX644015	–	
	*Platynereis dumerilii*	–	AF178678	–	
Nephtyidae	*Nephtys *sp.	–	EU293739	–	
Syllidae	*Eusyllis blomstrandi*	–	NC031402	–	

Newly sequenced taxa are in bold.

Trees calculated using amino acid sequences often recover different topologies than those strictly estimated from nucleotide sequences, as the former are often more conservative. In order to account for this as a potential source of phylogenetic uncertainty, we analysed four separate datasets: 13 PCG translated into amino acids (AA) + 12S rRNA + 16S rRNA; 13 PCG translated into AA + 12S rRNA + 16S rRNA + 18S rRNA + 28S rRNA; 13 PCG (nucleotides) + 12S rRNA + 16S rRNA; 13 PCG (nucleotides) + 12S rRNA + 16S rRNA + 18S rRNA + 28S rRNA. All datasets were concatenated using Sequence Matrix^72^.

Phylogenetic reconstructions for each dataset were performed using Bayesian and maximum likelihood (ML) methods. Best-fit gene partitions were selected using the option ‘-m TESTONLYMERGE’^73^ in ModelFinder^74^ as implemented in IQ-Tree v.1.6.12. Model selection was based on the Bayesian Information Criterion (BIC). Model MtZoa with gamma distribution and a proportion of invariable sites (MtZoa + I + Γ) was optimal for the AA translated PGCs, while a GTR model with gamma distribution and a proportion of invariable sites (GTR + I + Γ) was the optimal model for nt-PGCs and all the rRNA markers. Bayesian analyses (BA) were performed using MrBayes v.3.2.6^75^. All datasets were submitted with two independent runs using four chains (three heated, one cold). Each chain was allowed to run for 30 million generations, with sampling set for every 1000 generations. The first 10 million generations were discarded as burnin. A 50% majority-rule consensus tree with posterior probabilities was constructed using the remaining trees after burnin. Convergence of all MCMC runs were verified using TRACER v.1.6.0^76^. Maximum likelihood analyses were computed using RAxML version 7.2.8^77^ with nodal support being estimated via non-parametric bootstrapping with 1000 replicates^78^. A general time reversible (GTR) model with corrections for a discrete gamma distribution (GTR + Γ) was specified for each partition as invariable sites is incorporated within the estimations. Transition matrices for amino acid partitions used the WAG model of protein evolution as it has been shown to be a superior alternative, estimating higher likelihoods than other commonly used models^79^.

Model selection analyses were run on “Hydra”, the Smithsonian Institution High Performance Cluster (SI/HPC), while phylogenetic trees were calculated on the CIPRES Science Gateway^80^. All data, including phylogenetic trees and R-scripts used throughout this study are publicly available on the Open Science Framework (OSF) repository (ID. https://osf.io/njy9z/).

### Hypotheses testing

With increased visualization of the ocean, observations suggest that scale worms (e.g., Polynoidae) are far more capable of swimming than previously recognized. Through body undulation and metachronal beating of their parapodia, they move efficiently and easily into the water column. This is the case for most species of the symbiotic genus *Branchipolynoe,* which despite being considered as a benthic parasitic clade, is known to actively swim between deep sea mussel bed^41^; “On videotape, the blood-red polynoids can be seen leaving their hosts, swimming freely, scurrying about and hiding among the clusters of mussels” (Pettibone^41^, p. 236). In addition, while rare, a select few polynoid genera maintain a permanent position within the water column, combining drifting, posturing and active swimming behaviours. Such drifting behaviours are currently only known from holopelagic and anchialine cave genera^40^.

Since nearly all scale worms are considered benthic, we expect both swimming and drifting to have evolved secondarily in *Branchipolynoe*, the holopelagic *Drieschia* and the anchialine cave genera *Gesiella* and *Pelagomacellicephala.* Both swimming and drifting may be correlated with strong morphological modifications. We expect correlations to exist between specific morphological traits and behavioural patterns associated with accessing the water column. Specifically, we hypothesize that active swimmers will have a greater ratio of parapodial length to width (referred to as “parapodial size”), and is doing so by increasing the surface area of the appendage, producing more power during strokes, making it easier for parapodia to reach into the water outside their boundary layer. In drifters, our hypothesis is that both the total cirri length and the overall body length will be larger than in purely benthic taxa, increasing the overall surface area and increasing buoyancy of the animal. Finally, we have also included the character state of eyes in our analyses as they play an integral role in the interactions between habitat and lifestyle, occurring in the holopelagic *Drieschia* and shallow water polynoids*,* yet missing in the deep sea and cave polynoids. While the evolution of eyes has already been investigated in detail in previous studies^40^, we include it here in order to identify relationships to different swimming behaviours.

Our goal was to identify putative adaptations across polynoid lineages, combining multiple comparative methods while addressing morphological traits and sequence substitution rates. Morphologically, our expectations are that species that access the water column more frequently will display morphological traits to minimize energy consumption, promoting positive buoyancy (i.e., longer dorsal cirri in relation to body size) while increasing efficiency of parapodial paddling and body undulation (i.e., longer parapodia in relation to parapodial width).

#### Character selection and coding

All character states were measured and coded from personal collections, original descriptions, recent literature or from scaled micrographs (see Supplementary Figure S3). Measurements for *Lepidonotus* sp. collected in Hong Kong (see Zhang et al.^25^) were coded from *L. tenuisetosus* (Gravier, 1902), as it is the common occurring species in Hong Kong. Characters for *Halosydna* sp. and *Melaenis* sp. were coded from the type species of each genus^81,82^. *Drieschia* cf. *elegans* was measured from scaled live images of the sequenced individual, as well as from unpublished drawings of *D. elegans* Seidler, 1924 collected from the Cabo Verde Exclusive Zone by Marian H. Pettibone. All measurements were done using ImageJ v.2.0.0. Models for each of the response variables were formulated as follows:

Parapodial morphology is highly variable across Polynoidae, suggesting that certain modifications, such as a larger size, may play a role in the ability of some species to access the water column. “Parapodial length” was measured from the neuropodial acicular lobe to the attachment along the body wall, while “parapodial width” was measured across the widest point of the parapodium, excluding branchia and dorsal and ventral cirrophores.

The dorsal cirri comprise two separate parts in scale worms, the sensory style which projects away from the body, and the muscular cirrophore to which the style is attached. In all analyses, the traits “style length”, “cirrophore length” and “cirri length” were included. The dorsal style was measured along its midline from the attachment point on the cirrophore to the outermost tip, while the dorsal cirrophore was measured along the midline from the style attachment point to the insertion of the cirrophore on the parapodia. We considered the length of the dorsal cirri as the combined lengths of both the style and cirrophore.

“Body length” and “body width” (excluding parapodia) were included in order to account for potential allometric relationship of certain body traits. Body length was measured from the terminal end of the prostomium to the terminal end of pygidium along the midline axis of the body, while body width was measured as the widest horizontal distance between the body walls.

#### Comparative analyses

In order to test our hypotheses, we first estimated the phylogenetic signal of each of the quantitative traits using indices of Pagel’s lambda^83–85^ and Bloomberg’s K^86,87^. Our expectations are that parapodial elongation (i.e., parapodial length, parapodial width) and the cirri ratio (cirri length: body length) will present a lower phylogenetic signal, reflected by low values of these indexes and in that these traits evolved several times and independently to the phylogeny.

Since Pagel’s lambda and Blomberg’s K indexes are highly susceptible to dataset size, which might affect their significance, we explicitly tested for correlation of each of the potentially adaptive traits towards different behavioural and ecological parameters using methods of phylogenetic generalized least squares (PGLS). We expect to find a positive relationship of the “cirri length” in holopelagic and anchialine cave species, independent of their body size, but not to parapodial size as all polynoids appear to use their parapodia in a similar manner to swim.

All continuous variables were tested for autocorrelation using Pearson correlation index. “Body length” and “body width” (R^2^ = 0.95), as well as “parapodial length” and “parapodial width” (R^2^ = 0.97) were highly correlated, so only length measurements from these two characters were included in further analyses. All other variables were not autocorrelated so were retained as ratios.

Phylogenetic signal was estimated on continuous characters with λ and *K* statistics as both indices compare the actual distribution and values of a given character on a phylogeny against the null hypothesis that the character follows a Brownian model of evolution^88^. Estimated λ and *K* statistics were implemented using the function “phylosig,” as implemented in the R package phytools v.0.5-38^89^ on the ML phylogenetic tree constructed with the 13 PCG (nucleotides) + 12S rRNA + 16S rRNA + 18S rRNA + 28S rRNA. Maximum likelihood trees incorporate branch lengths as total units (vs. relative in ultrametric) from a single (vs. a sample of trees in Bayesian) phylogenetic optimization. In order to verify that our results are robust upon tree generating parameters, we ran λ and *K* statistics on both ML non-ultrametric and ML ultrametric trees converted to ultrametric using the function “chronos” implemented in the R package ape v 5.3^90^. We report p values for both statistics despite expecting a generally low significance in our analyses due to low sample size and relatively large divergence times. We then showed the evolution of these continuous characters on our phylogeny using the function “contMap” in the R package phytools^89^. In order to estimate the phylogenetic information of discrete characters, we performed ML ancestral character reconstructions on our maximum likelihood phylogeny by implementing the function “ace”, which is incorporated in the R package ape v.3.5^91^.

Once we estimated the phylogenetic signal for each trait, we calculated their correlation to the behavioural traits (i.e., “swimming” and “drifting”) using phylogenetic general least squares models (PGLS), which are extensions of general linear models that account for the phylogenetic structure of the residuals^92^. The variables “cirri length” and “parapodial length” were selected as response variables, as they were showing low phylogenetic signal. The variable “swimming” and “drifting” (two levels: absent/present) were chosen as explanatory variables (see Table 3). All PGLS analyses were run using the function ‘pgls’ included in the R package caper v.1.0.1^93,94^.

In order to further understand the morphological changes in polynoids related to different behavioural patterns associated with accessing the water column, we also tested the relationship of the remaining characters (e.g., “style length”, “cirrophore length”, “cirri length” (cirrophore + style), “parapodial length”) to both “swimming” and “drifting” (see Table 3). In those analyses, the continuous variable “body length” was also included as an explanatory variable to account for the potential effect of allometric growth on our results (i.e., larger animals are expected to have larger cirri). The analyses were performed as indicated above and the results are included on the Open Science Framework (OSF) repository (ID. https://osf.io/njy9z/).

## Supplementary Information

Supplementary Information 1.

Supplementary Information 2.

## Data Availability

Newly obtained sequences were deposited in GenBank Repositories under accession numbers listed in Table 4. All data, including phylogenetic trees and R-scripts used throughout this study are publicly available on the Open Science Framework (OSF) repository (ID. https://osf.io/njy9z/).
