# Supplementary material for: Morphological convergence and adaptation in cave and pelagic scale worms (Polynoidae, Annelida)
Source: Sci Rep. 2021 May 21;11:10718. doi: 10.1038/s41598-021-89459-y (PMC8139957; doi:10.1038/s41598-021-89459-y)
Supplement: Supplementary file 2 — Supplementary Information 2. [file 41598_2021_89459_MOESM2_ESM.pdf]

Morphological convergence and adaptation in cave and pelagic scale worms (Polynoidae, Annelida)

Brett C. Gonzalez[1*], Alejandro Martínez[2], Katrine Worsaae[3], Karen J. Osborn[1, 4]

[1] Smithsonian Institution, National Museum of Natural History, Department of Invertebrate Zoology, P.O. Box 37012, Washington D.C., USA

[2] Molecular Ecology Group (MEG), Water Research Institute (IRSA), National Research Council of Italy (CNR), Largo Tonolli, 50, 28922. Pallanza, Italy

[3] Marine Biological Section, Department of Biology, University of Copenhagen, Universitetsparken 4, Copenhagen Ø, Denmark

[4] Monterey Bay Aquarium Research Institute, 7700 Sandholdt Road, Moss Landing, CA 95039 USA

[*] gonzalezb@si.edu

Supplementary Figure 1

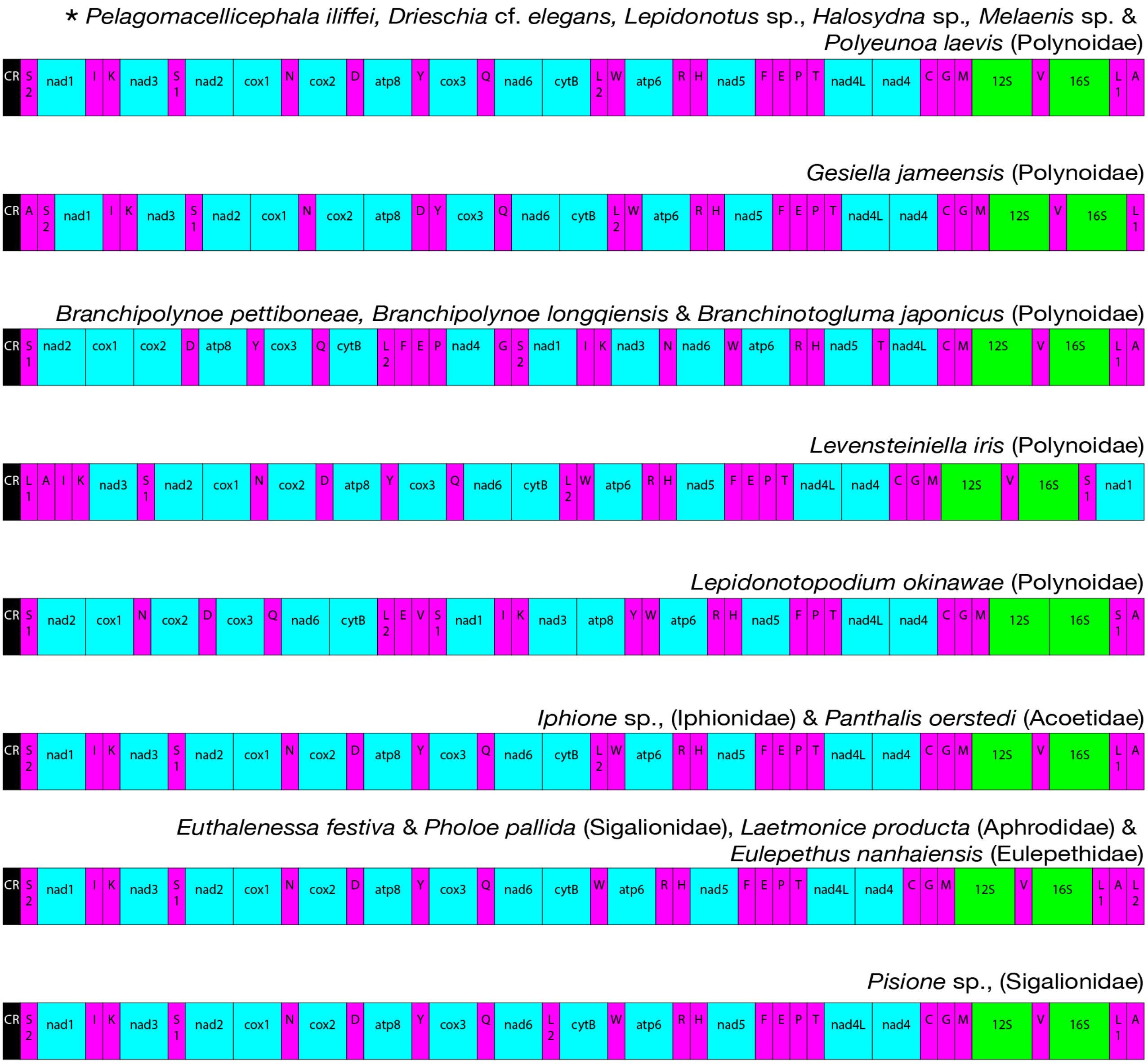

Gene orders across available Aphroditiformia mitogenomes. Asterisk (*) denotes putative Polynoidae gene order ground pattern. Likely location of the control region (CR) is indicated by black filled rectangles, while fuchsia rectangles indicate tRNA's, turquoise for PCGs and green for mt rRNAs. Gene orders taken and redrawn from Zhang et al. (2018).

Supplementary Figure 2

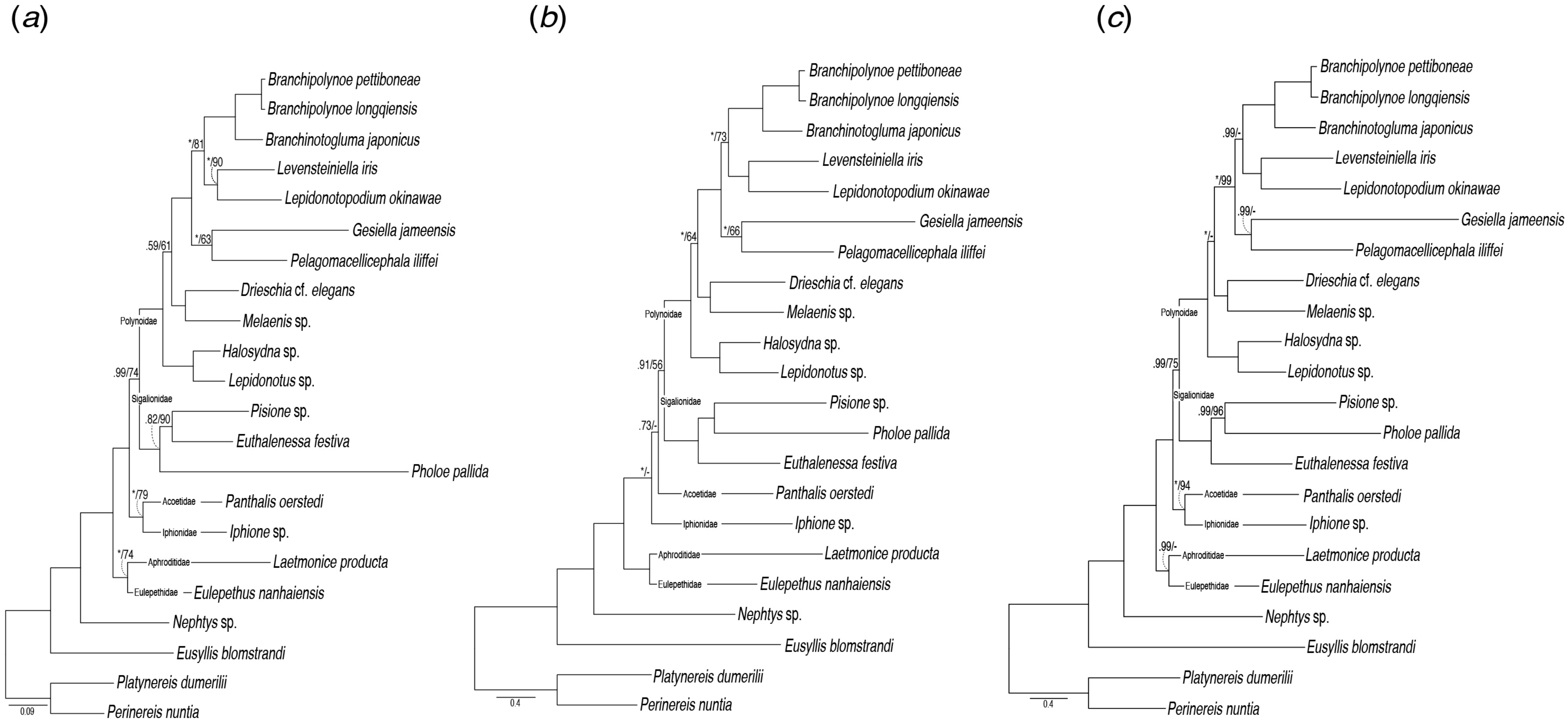

Mitogenomic analyses of Aphroditiformia. (*a*) Mitogenome relationships of Aphroditiformia based on the concatenated dataset of 13 protein coding genes translated into AA (amino acids) + 12S + 16S. (*b*) Mitogenome relationships of Aphroditiformia based on the concatenated dataset of 13 protein coding genes, Nuc (nucleotides) + 12S + 16S + 18S + 28S. (*c*) Mitogenome relationships of Aphroditiformia based on the concatenated dataset of 13 protein coding genes, Nuc + 12S + 16S. All tree topologies based on the Bayesian analysis (BA) of the dataset. All nodes are fully supported unless marked. Nodes not recovered or with support lower than BPP = 0.5 and MLB = 50 are represented by a dash (-). Asterisks (*) denote BPP = 1.0 or MLB = 100.

Supplementary Figure 3

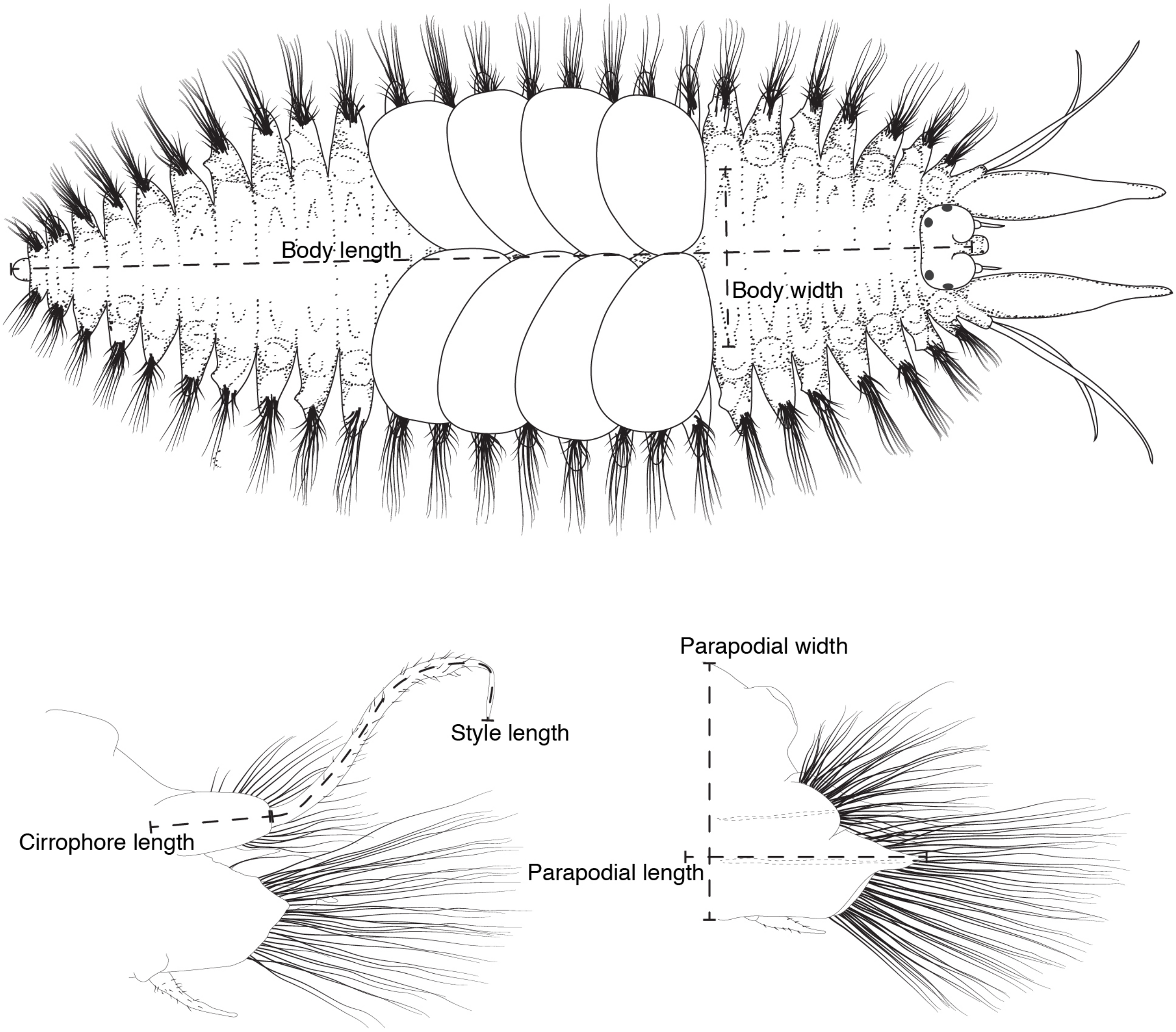

Illustrative guide from where each of the measurements for the hypothesis testing were made. Dashed line represents measured pathways. Images not to scale or representative of species used. All measurements were made using ImageJ v.2.0.0. Images redrawn from the Pettibone collections by KJO as part of the Osborn Lab-NMNH.
